# Advances in Screening and Development of Therapeutic Aptamers Against Cancer Cells

**DOI:** 10.3389/fcell.2021.662791

**Published:** 2021-05-19

**Authors:** Zheng Li, Xuekun Fu, Jie Huang, Peiyuan Zeng, Yuhong Huang, Xinxin Chen, Chao Liang

**Affiliations:** ^1^Department of Biology, Southern University of Science and Technology, Shenzhen, China; ^2^Department of Biochemistry, University of Victoria, Victoria, BC, Canada

**Keywords:** aptamer, cancer cells, targeted therapy, SELEX, clinical application

## Abstract

Cancer has become the leading cause of death in recent years. As great advances in medical treatment, emerging therapies of various cancers have been developed. Current treatments include surgery, radiotherapy, chemotherapy, immunotherapy, and targeted therapy. Aptamers are synthetic ssDNA or RNA. They can bind tightly to target molecules due to their unique tertiary structure. It is easy for aptamers to be screened, synthesized, programmed, and chemically modified. Aptamers are emerging targeted drugs that hold great potentials, called therapeutic aptamers. There are few types of therapeutic aptamers that have already been approved by the US Food and Drug Administration (FDA) for disease treatment. Now more and more therapeutic aptamers are in the stage of preclinical research or clinical trials. This review summarized the screening and development of therapeutic aptamers against different types of cancer cells.

## Introduction

Aptamers initially were described by researchers in 1990 (Ellington and Szostak, 1990). They are short-chain nucleotide sequences, generally DNA or RNA of 20 to 100 nucleotides. With a unique tertiary structure, aptamers specifically bind to target molecules (Röthlisberger and Hollenstein, 2018). Aptamers are reproducible and programmable. Enzymatic degradation is resisted by the chemical modification of aptamers. The chemical integrity and bioavailability of aptamers are ensured through optimization under the physiological condition (Mallikaratchy et al., 2006; Zhu and Chen, 2018). When aptamers are modified with a hydrophobic group, they have an excellent binding affinity (Dunn et al., 2017; Odeh et al., 2020). An outstanding method, Systematic Evolution of Ligands by Exponential Enrichment (SELEX), was used to screen aptamers (Dunn et al., 2017). The target of aptamers range from small molecules (Li and Liu) to biomacromolecules (Mallikaratchy et al., 2006), infected cells (Liu et al., 2019), stem cells (Hou et al., 2015; Gang et al., 2017), and cancer cells (Moosavian and Sahebkar, 2019; Kordasht and Hasanzadeh, 2020).

For most drugs, there are two problems in the treatment of tumors, the first one is weak therapeutic effects and another is strong off-target toxicity (Lammers et al., 2012). Therefore, improving the effectiveness of drugs and reducing side effects are major challenges faced by researchers (Moosavian and Sahebkar, 2019). Selecting specific ligands that target tumors is the most widely used strategy in targeted therapy. The specific ligand has almost no affinity for normal cells. Besides, it does not produce any toxic effects (Pérez-Herrero and Fernández-Medarde, 2015). Now various ligands, antibodies, antibody fragments, peptides, nucleic acids, and small molecules, have been used for this purpose (Dougan and Dougan, 2017). The conjugation of specific ligands and drugs enhances the internalization of encapsulated drugs and the accumulation of drugs in tumors (Alavi and Hamidi, 2019). Among these ligands, aptamers are considered the most suitable ligands for active tumor targeting. Due to high specificity, aptamers have become the subject of extensive research during the past decades as targeting ligands. Aptamers with great affinity and selectivity can be modified, including direct conjugation with drugs and drug-encapsulated nanoparticles. In addition, aptamer holds stable under a broad range of physical and chemical conditions. They can be released after the drug is transported to target cells (Soldevilla et al., 2018; Zhu and Chen, 2018).

Apart from being used as targeting ligands, aptamers also act as small molecular agents, which means that they inhibit the functions of target proteins (Gelinas et al., 2016; Adachi and Nakamura, 2019). So far, many therapeutic aptamers are in preclinical or clinical development (Adachi and Nakamura, 2019). Aptamers act as antagonists to impede the interaction of tumor-related targets (proteins or receptor-ligands) or act as agonists to activate the function of anti-cancer target receptors to achieve the purpose of cancer treatment. In the treatment of various diseases, including eye diseases, cardiovascular diseases, tumors, and inflammations, aptamers have entered clinical trials as therapeutic drugs. Pegaptanib is the only therapeutic aptamer approved by the FDA for the treatment of age-related macular degeneration (Ng et al.). The aptamers used in the treatment of cardiovascular diseases and inflammation are all in the first or second phase of clinical trials to evaluate the efficacy and safety without further updates (Maasch et al., 2008; Waters et al., 2009; Povsic et al., 2014). At present, there are three types of aptamers completed clinical trials for tumor treatment. The first one is AS1411, which binds to the external domain of the target (nucleolin) with high affinity. In various clinical cancer models, including lung cancer, breast cancer, kidney cancer and liver cancer, etc., AS1411 shows the ability to inhibit cell proliferation and to induce cell apoptosis (Yazdian-Robati et al., 2019). The second one is NOX-A12, which is an L-type RNA aptamer, so it can resist nuclease degradation. It is an antagonist of chemokine (C-X-C motif) ligand 12 (CXCL-12), which inhibits tumor cell proliferation, new blood vessel formation, and metastasis (Duda et al., 2011). Besides, AGRO100 is also an aptamer that binds to nucleolin to produce anti-proliferation effects in tumor cells. In some phase I clinical trials, AGRO100 showed high safety and anti-tumor proliferation effects (Laber et al., 2004). So far, aptamers have not been approved as clinical drugs for the treatment of tumors. Compared with antibodies, the development of therapeutic aptamers is relatively slow (Zhou and Rossi, 2017). There are still challenges in developing aptamers into tumor therapeutics. The success of aptamers as therapeutic drugs in the future requires overcoming the challenges and fully developing unique properties.

## Properties of Aptamers

Due to the flexibility of short-chain nucleotides, aptamers have unique chemical properties. In general, aptamers fold into stem, loop, bugle, pseudoknot, G-quadruplex, and kissing hairpin structures, and further fold to form more complex tertiary structure. Complex folding allows aptamer to specifically recognize the target (Gelinas et al., 2016; Zhou and Rossi, 2017). A broad range of targets includes organic molecules (Umar and Kit, 2020), proteins (Umar and Kit, 2020), nucleotides (Yue et al., 2011), cancer cells (Guizhi and Xiaoyuan, 2018), bacteria (Alizadeh et al., 2018), toxins (Frohnmeyer et al., 2019), or viruses (Cesewski and Johnson, 2020). Compared with antibodies, aptamers with flexible structures and very small molecular weights can cross the blood-brain barrier (Cheng et al., 2013), a physiological barrier that antibodies cannot enter, and bind to target molecules with high affinity and specificity. Aptamers with unique properties are combined with targets to directly exert the efficacy of drugs to treat cancer. At the same time, aptamers act as carriers for drug delivery, combined with drugs to achieve targeted drug delivery, and play an important role in targeted tumor therapy.

Many therapeutic antibodies have been used clinically. Aptamers have many advantages over antibodies (Table 1). Aptamers are produced by chemical synthesis *in vitro*, which have great benefits including short synthesis time, low cost, high stability, and specificity (Zhang Y. et al., 2019). The small and flexible structure of aptamers allows them to bind with smaller targets or hide binding domains that some antibodies cannot access (Zhou and Rossi, 2017). However, disadvantages of aptamers are metabolic instability (Lakhin et al., 2013) and rapid kidney filtration (Guo, 2011). Therefore, methods of chemical modifications and conjugations have been developed to overcome the disadvantages. Despite these limitations, researchers have learned lessons from the clinical development and application of nucleic acids, and continue to pursue the development of therapeutic aptamers (Yoshihiro et al., 2018).

**TABLE 1:** Comparison of aptamers and antibodies.

	**Aptamer**	**Antibodies**
Synthesis	*In vitro*, SELEX	Produced *in vivo*
Target potential	Can target any small molecules	Difficult to raise antibodies to toxins (not tolerated by an animal) or non-immunogenic targets
Stability	Stable at room temperature.	Must be refrigerated for storage and transport
Affinity	High and increased in multivalent aptamers.	Dependent on the number of epitopes on the antigen
Specificity	Single point mutations identifiable.	Different antibodies might bind the same antigen
Activity	Uniform activity regardless of batch synthesis	The activity of antibodies varies from batch to batch
Modifiability	Wide variety of chemical modifications to molecule for diverse functions	Limited modifications to molecule
Immunogenicity	No immunogenicity	Significant immunogenicity
Shelf life	Unlimited shelf life	Limited shelf life
Tissue uptake/kidney filtration	Fast	Slow

## Production of Aptamers

To select high-affinity aptamers for targets, SELEX is a mature and widely used technology. Researchers have developed different SELEX methods to meet different experimental needs. These methods include capillary electrophoresis-SELEX (CE-SELEX) (Zhu et al., 2019), high-throughput (HT)-SELEX (Kato et al., 2020), microfluidic SELEX (Sinha et al., 2018), and cell SELEX (Harleen and Kaur, 2018).

**FIGURE 1:**
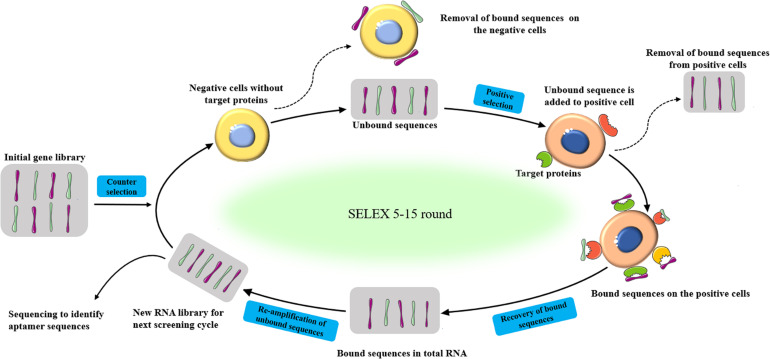
Schematic representation of the SELEX procedure to produced aptamers for specific target biomolecules.

The basic process of SELEX is a four-step repeated cycles of incubation, combination, distribution, and amplification (Figure 1). Firstly, a random oligonucleotide sequence library (starting library) is incubated with the target molecules. During the incubation process, some sequences bind to the target molecule, while others bind weakly or do not react with the target. Then, the bound sequence is separated from the weakly bound or unbound sequence. After that, Polymerase chain reaction (PCR) (DNA sequences) and reverse transcription PCR (RT-PCR) (RNA sequences) were adopted to amplifying eluted oligonucleotides to enrich the library. Various methods have been developed to generate ssDNA from the resulting double-stranded DNA (dsDNA), including asymmetric PCR (Citartan et al., 2012), denaturing urea-polyacrylamide gel (Ram et al., 2017), lambda exonuclease, and T7 gene 6 exonuclease digestion (Subramanian et al., 2003; Citartan et al., 2011), and magnetic separation by beads coated with streptavidin (Espelund et al., 1990). The obtained ssDNA is then used in the next round of selection (Bayat et al., 2018). The process of binding, separation, amplification, and pool conditioning needs to be repeated before the resulting nucleic acid pool is enriched enough with target-binding sequences (Komarova and Kuznetsov, 2019). In general, researchers get a rich pool through 5–15 rounds of selection. The enriched pool is sequenced and bioinformatics analysis is used to identify aptamer candidates from the sequencing results (Lewis et al., 2017). The selected aptamer is chemically synthesized and characterized (Wang et al., 2018).

## Screening and Development of Therapeutic Aptamers Against Cancer Cells

In the past few decades, many specific aptamers have been developed for tumor treatment (Table 2), such as breast cancer (Liu et al., 2017), colorectal adenocarcinoma (Chen et al., 2017), lung cancer (2018), liver cancer (Zhang G.Q. et al., 2019), prostate cancer (Gray et al., 2018), leukemia (Tan et al., 2019), renal cell carcinoma (Zhang et al., 2018), oral cancer (Simmons et al., 2014), cervical cancer (Carvalho et al., 2019), bladder cancer (Yao et al., 2020), stomach cancer (Ding et al., 2015) and multiple myeloma (Waldschmidt et al., 2017).

**TABLE 2:** Examples of aptamers with potential as targeted drugs.

**Cancer types**	**Aptamers**	**Targets**	**Clinical applications**	**Cell lines**	**References**
Breast cancer	PNDA-3	Periostin	None	MCF7, MDA-MB-231cell	Lee et al., 2013
	HeA2-3	HER2	None	SKBR3, MDA-MB-231, SKOV3 cell	Gijs et al., 2016
	ApMNK2F	MNK	None	MDA-MB-231, MCF7 cell	García-Recio et al., 2016
	ApMNK3R				
Colorectal cancer	YJ-1	CEA	None	LS174T,SW480 cell	Lee et al., 2012
	Aptamer	PDGF-BB	None	Caco-2, SW480 cell	Sae-Lim et al., 2019
	MP7	PD-1	None	MC38 cell	Prodeus et al., 2015
Lung cancer	AptPD-L1	PD-1	None	CT26, LL/2 cell	Lai et al., 2016
	AP-74 M-545	Gal-1	None	LL/2 cell	Tsai et al., 2019
	TBA535.	Hrombin	None	Calu-6 cell	Esposito et al., 2018
Liver cancer	AS1411	HCC	None	Hepatocellular carcinoma cell	Cho et al., 2016
	CL-4RNV616	EGFR	None	Huh-7 cell	Wang et al., 2019
Prostate cancer	Apt63	Ecto-ATP5B	None	PC-3, RWPE-1, MCF10, 67NR, E0771 cell	Speransky et al., 2019
	A9g	PSMA	None	PC-3 cell	Dassie et al., 2014
	AGRO100	Nucleolin	I clinical trial	Prostate cancer cell	Laber et al., 2005
LeukemiaLeukemia	AS1411	Nucleolin	None	MV4-11 cell	Soundararajan et al., 2009
	β-arr2As	β-arrestin	None	K562 cell	Kotula et al., 2014
Renal cell carcinoma	SW-4	ccRCC	None	786-O cell	Zhang et al., 2018
	AS1411	Nucleolin	II clinical trial	Renal cell carcinoma	Rosenberg et al., 2014
Oral cancer	Aptamer	Heparanase	None	HSC-3 cell	Simmons et al., 2014
Cervical cancer	A2	E7	None	SiHa, CaSki, HeLa cell	Clare et al., 2013
Bladder cancer	Aptamer	β-catenin	None	SW780, 5637 cell	Xie et al., 2018
Stomach cancer	Aptamer	HER2	None	N87 cell	Mahlknecht et al., 2013
Multiple myeloma	NOX-A12	CXCL12	I clinical trial	U266, NCI-H929 cell	Waldschmidt et al., 2017

### Therapeutic Aptamers Against Breast Cancer Cells

Abnormal expression of human epidermal growth factor receptor 2 (HER2, also known as ErbB2) is present in 15–20% of all breast cancers (Waks and Winer, 2019). It is a molecular target with the potential for targeted cancer treatment. Gijs et al. screened the aptamer HeA2 through the SELEX method and identified two subtypes HeA2-1 and HeA2-3 by next-generation sequencing and bioinformatics methods. HeA2 binds to HER2 protein with high specificity. Interestingly, the authors’ results proved HeA2-3 was internalized into breast cancer cells and had an inhibitory effect on the growth and survival of breast cancer cells (Gijs et al., 2016). Periostin is a member of the extracellular matrix (ECM) protein. In tumor progression, its overexpression regulates the tumor microenvironment and affects the proliferation, invasion, and migration of tumor cells (Kudo, 2007). Lee et al. screened benzyl-d(U)TP modified DNA aptamers (PNDA-3) that are specifically bound to human periostin and characterized the function of the aptamers in breast cancer progression. PNDA-3 significantly inhibited the metastasis and growth of periostin-positive breast cancer. In the 4T1 orthotopic mouse model, the administration of PNDA-3 appreciably reduced the growth and distant metastasis of the primary tumor (Lee et al., 2013). MAP kinase-interacting kinase (MNK) is activated through the MAP kinase pathway and phosphorylates eukaryotic translation initiation factor 4E (eIF4E) at a single site. EIF4E and its phosphorylation are vital parts of cancer and tumorigenesis (Tian, 2017). García-Recio et al. (2016) screened two aptamers, called apMNK2F and apMNK3R. The selected aptamers were highly specific for MNK1. ApMNK2F and apMNK3R bind with MNK1 to produced significant translational inhibition, inhibiting tumor cell proliferation, migration, and colony formation in MDA-MB231 breast cancer cells (García-Recio et al., 2016).

### Therapeutic Aptamers Against Colorectal Cancer Cells

Carcinoembryonic antigen (CEA, CEACAM5, or CD66e) is overexpressed in most cancers. It plays an important role in inducing tumor metastasis (Hammarström, 1999; Taheri et al., 2000). Liver metastasis is the main cause of death in colorectal cancer patients. Inhibiting liver metastasis is an effective way to improve the survival rate of patients with colorectal cancer (Tauriello et al., 2017). Lee et al. designed an RNA aptamer (YJ-1) that is specifically bound to CEA-positive cells. The homotype accumulation, migration, and invasion of CEA-positive cancer cells were inhibited by YJ-1. YJ-1 induced apoptosis of colon cancer cells by blocking the interaction between death receptor 5 and CEA. The transfer of human colon cancer cells to the liver was prevented by YJ-1 in the mouse experiments (Lee et al., 2012). Overexpression of platelet-derived growth factor-BB (PDGF-BB) is related to colorectal carcinogenesis. Sae-Lim et al. (2019) developed a DNA aptamer that is specifically bound to PDGF-BB. This DNA aptamer interfered with the binding of PDGF-BB and its receptor and inhibited the proliferation of colorectal cancer (CRC) cells by down-regulating the Ras/Raf/MEK/ERK signaling pathway (Sae-Lim et al., 2019). Programmed cell death-1 (PD-1, Pdcd1) negatively regulates antigen receptor signaling. It belongs to an immunoreceptor which is a family member of CD28/CTLA-4. The engagement of PD-1/PD-L1 suppresses T cell function and the axis is considered one of the major pathways involved in tumor immune evasion (Okazaki and Honjo, 2007). Proteus et al. synthesized a DNA aptamer (MP7), which mimicked an antibody that specifically bound to PD-1 and blocked the binding of PD-1 and PD-L1. The pegylated form of MP7 inhibited the growth of colon cancer in PD-L1-positive mice *in vivo* (Prodeus et al., 2015).

### Therapeutic Aptamers Against Lung Cancer Cells

Immunotherapy has dramatically improved the survival rate of some lung cancer patients (Yu et al., 2016). Lai et al. (2016) developed a new type of PD-L1 antagonistic DNA aptamer (aptPD-L1). In CT26 and LL/2 murine syngeneic tumor models, aptPD-L1 had no direct cytotoxicity to cancer cells, but induced expression of IFNγ-inducible chemokines CXCL9/10 which regulated the tumor microenvironment. AptPD-L1 helped the recovery of T cell function and worked together with cytokines to resist tumor growth (Lai et al., 2016). Galectin-1 (Gal-1), which is related to several important biological processes in the development of tumors. Gal-1 has immunosuppressive effects on tumors by directly promoting T cell apoptosis or indirectly damaging the differentiation of tumor cells and T cells in their microenvironment (Pace et al., 1999). A DNA aptamer (AP-74 M-545) was developed for Galectin-1. The researchers developed a mouse lung cancer model to evaluate the characteristics, functions, and effects of AP-74 M-545. Aptamer blocked the interaction between Gal-1 and CD45 to inhibit T cells from apoptosis and restored T cell-mediated immunity. AP-74 M-545 exerted anti-tumor effect by restoring the immune function of mice (Tsai et al., 2019). The thrombin binding aptamer (TBA) has anticoagulant and antiproliferative effects. Esposito et al. (2018) chemically modified TBA to obtain two oligonucleotides, TBA353 and TBA535. Those two aptamers had significant anti-proliferative activity against lung cancer Calu-6 cells, while TBA535 inhibited cancer cell movement, indicating that it inhibited cell metastasis or tumor invasion (Esposito et al., 2018).

### Therapeutic Aptamers Against Liver Cancer Cells

AS1411 is a DNA aptamer with 26 nucleotide that forms a guanine quadruplex structure. AS1411 binding to nucleolin that is aberrantly expressed on the cell membrane of many tumors. Nucleolin is involved in cell adhesion, division, and migration. AS1411 binds with nucleolin and inhibits tumor cell growth (Trinh et al., 2015). Cho et al. (2016) evaluated the affinity and specificity of the modified AS1411 to hepatocellular carcinoma cells (HCC). Besides, the authors studied the therapeutic potential of the modified aptamer in liver cancer. Experimental data showed that the modified AS1411 significantly reduced the proliferation of HCC cells *in vitro*. Microarray analysis showed that the modified AS1411-aptamer inhibited the growth of HCC cells by up-regulating the expression of galectin-14 (Cho et al., 2016). Epidermal growth factor receptor (EGFR) is a member of the ErbB receptor family, *in vivo* EGFR expression is associated with the progression of a variety of cancers, including breast cancer, glioma, lung cancer, and liver cancer. Inhibition of EGFR limits the growth and proliferation of EGFR-positive cancers. Wang et al. (2019) investigated a 27mer aptamer with therapeutic potential. It is CL-4RNV616 that contains 2′-O-Methyl RNA and DNA nucleotides and specifically targets EGFR. The researchers found CL-4RNV616 not only effectively recognized and inhibited the proliferation of Huh-7 liver cancer, MDA-MB-231 breast cancer, and U87MG glioblastoma cells but also effectively induced cancer cell apoptosis (Wang et al., 2019).

### Therapeutic Aptamers Against Prostate Cancer Cells

Speransky et al. (2019) designed an RNA aptamer (Apt63) to distinguish prostate cancer cell lines with high metastatic potential from low metastatic potential. Apt63 was not bound to non-metastatic cancer cells but stuck to the beta subunit of F1Fo ATP synthase (ATP5B), which existed in the plasma membrane of cancer cells. The binding of Apt63 and plasma membrane-located ATP synthase (Ecto-ATP5B) could destroy the basic survival mechanism of tumors with high metastatic potential and lead to rapid cell death (Speransky et al., 2019). Prostate-specific membrane antigen (PSMA) is a glycosylated type II membrane protein. In the process of malignant transformation, prostate cancer (PC) has an abnormally high level of PSMA on the cell surface. PSMA represents an ideal target for the diagnosis and treatment of PC (Haberkorn et al., 2016; Virgolini et al., 2018). Dassie et al. (2014) described the therapeutic potential of an RNA aptamer (A9g). A9g was specific for PC cells expressing PSMA and had a therapeutic effect on advanced PC by inhibiting the enzymatic activity of PSMA. AGRO100 was an oligonucleotide that bound to nucleolin. The combination of A9g and AGRO100 led to a strong anti-proliferative response in tumor cells. Laber et al. (2005) conducted a phase I clinical trial of the aptamer AGRO100. There are 17 patients with advanced cancer were enrolled, including prostate cancer patients. Patients who receive AGRO100 therapy were in stable condition and had no adverse immune reactions (Laber et al., 2005).

### Therapeutic Aptamers Against Leukemia Cells

Compared with normal cells, nucleolin is an overexpressed protein in the cytoplasm and plasma membrane of some tumor cells. Soundararajan et al. (2009) found that nucleolin in MV4-11 cells is the functional receptor of AS1411. In clinical trials, AS1411 not only shows promising anti-tumor activity but also has low serious systemic toxicity (Soundararajan et al., 2009). β-arrestin is a cell scaffold protein that promotes tumorigenesis in various tumor models. Kotula et al. (2014) adopted an interesting delivery strategy. DNA “targeted aptamers” directly delivered RNA “therapeutic aptamers.” Nucleolin-specific DNA delivery aptamers were coupled with a therapeutic aptamer targeting β-arrestin2, and the therapeutic aptamer was delivered to leukemia cells. Inhibition of β-arrestin2 led to the obstruction of multiple β-arrestin-mediated signaling pathways required for the progression of chronic myeloid leukemia (CML) (Kotula et al., 2014).

### Therapeutic Aptamers Against Renal Cell Carcinoma Cells

Zhang et al. identified the aptamer SW-4 targeting human renal clear cell adenocarcinoma cell 786-O from a library of known sequences. Further studies showed that aptamers were internalized into target cells in a temperature-dependent manner through caveolae-mediated endocytosis and accumulated at tumor sites. SW-4b inhibited the proliferation of 786-O cells by preventing the progression of the cell cycle in the S phase (Zhang et al., 2018). AS1411 has been completed in clinical trials. The safety of clinical treatment effect is excellent. However, the overall remission efficiency is low (Bates et al., 2017). It showed anti-tumor activity in a phase I study in patients with renal cell carcinoma, and no dose-limiting toxicity was observed (Miller et al., 2006; Stuart et al., 2009). Rosenberg et al. (2014) conducted a phase II study. The results were unsatisfactory. Continuous infusion of a dose of 40 mg/kg/day would not cause any serious side effects. AS1411 existed a lower cancer remission rate. No response was found in other patients, and the poor pharmacology and low potency of this unmodified DNA may limit its future development in unscreened cancer patients (Rosenberg et al., 2014).

### Therapeutic Aptamers Against Oral Cancer Cells

Heparanase is a β-1,4-endoglycosidase, which regulates the degradation and remodeling of extracellular matrix (ECM) (Hulett et al., 1999). It has been found that elevated levels of heparanase mRNA and protein in cancer patients have significantly reduced the postoperative survival time. The expression level of heparanase in cells is related to the metastatic potential of tumors (Koliopanos et al., 2001). Simmons et al. (2014) proved that the heparinase-specific aptamers inhibited tissue invasion of cells, and at the same time, aptamer was stable under physiological conditions and had no cytotoxicity to play a role in the treatment of oral cancer.

### Therapeutic Aptamers Against Cervical Cancer Cells

Human papillomavirus (HPV) is a DNA virus that is closely related to the cervical cancer. HPV16, a high-risk subtype, infects epithelial cells and leads to the development of cancer through viral oncogenes E6 and E7 (Bernard et al., 2010). Studies showed that E6 promoted the degradation of the tumor suppressor gene p53 (Lechner and Laimins, 1994), and E7 led to dysregulation of the S phase of the cell cycle (Nicol et al., 2011). Clare et al. (2013) discovered an RNA aptamer (A2) that had a high affinity for E7. A2 bound to the N-terminal residue of E7, which interacted with the cell cycle control protein pRb. In cervical cancer cell lines, A2 was able to induce apoptosis, and inhibit cell proliferation.

### Therapeutic Aptamers Against Bladder Cancer Cells

Transcription factors (TFs) in the cell nucleus play a crucial role in the process of cell gene expression. During the development of cancer, TF regulated the expression levels of oncogenes, tumor suppressor genes, and cell cycle-related molecules, thereby affected tumor formation, evolution, and metastasis (Haberle and Stark, 2018; Lambert et al., 2018). Xie et al. (2018) linked TF-specific RNA aptamers with targeted oncogenic miRNAs to form artificial long non-coding RNA (alncRNA). The transcription activity of TF was inhibited by alncRNA, and the transcription of oncogenes was controlled. The experiment showed that alncRNA in bladder cancer cell lines (5637, SW780) reduced the expression of TF target genes, cell proliferation and migration, and cell apoptosis was increased (Xie et al., 2018). Compared with the CRISPR/Cas system, alncRNA had both transcriptional regulation and post-transcriptional regulation of bladder cancer cells, showing a higher inhibitory effect in terms of the cell phenotype (Yao et al., 2020).

### Therapeutic Aptamers Against Stomach Cancer Cells

ErbB-2/HER2 has a high expression in gastric cancer, so it is used as a therapeutic target. For stomach cancer, Mahlknecht et al. (2013) screened an aptamer from the library. They found this aptamer was able to bind ErbB-2/HER2 specifically. Only when the aptamer formed trimers, it could play a therapeutic role. To be more specific, the complex contained trimeric aptamer and ErbB-2/HER2 entered the cytoplasm through endocytosis, then lysosomes hydrolyzed this complex. As a result, the growth of gastric cancer cells and the growth rate of tumors were blocked and reduced respectively (Mahlknecht et al., 2013).

### Therapeutic Aptamers Against Multiple Myeloma Cells

CXCL12 (Chemokine (C-X-C motif) ligand 12) is a chemokine, which is mainly related to cell trafficking and adhesion. Chemokines bind to specific G-protein-coupled seven-span transmembrane receptors. The binding of CXCL12 to its receptor CXC receptor 4 (CXCR4; CD184) will affect cell chemotaxis, cell survival, proliferation, and gene transcription (Teicher and Fricker, 2010). Waldschmidt et al. (2017) found that in multiple myeloma (MM), inhibition of cell adhesion-mediated drug resistance (CAM-DR) caused by bone marrow (BM) is the key to anti-myeloma treatment. NOX-A12, an L:-enantiomeric RNA oligonucleotide, is a specific inhibitor of CXCL12 (Sayyed et al., 2009). NOX-A12 functionally interfered with MM chemotaxis to the BM, caused multiple myeloma cells to be re-sensitized to therapeutic drugs. Aptamer NOX-A12 had anti-myeloma CAM-DR activity (Waldschmidt et al., 2017).

## Conclusion

Aptamers have been widely applied to the treatment of diseases. They not only be used alone as therapeutics but also be combined with drugs covalently/non-covalently to achieve targeted drug delivery. With excellent binding affinity and specificity, aptamers have been more and more widely developed and applied to diagnosis, analytics, bio-imaging, and aptasensors of diseases (Ştefan et al., 2021). Among many application areas of aptamers, tumor therapy has aroused great interest. Researchers are committed to developing new aptamers as drugs to inhibit cancer progression. This article summarizes the aptamers screened for tumor cells in different cancer types. These aptamers require further animal experiments and clinical trials to confirm subsequent drug development.

Currently, the development of therapeutic aptamers is relatively slow, and there is no aptamer as a medicine to treat cancer clinically. Only a few aptamers had undergone clinical trials and they also have not been successfully used in the treatment of tumors. The underdevelopment may be mainly related to the following reasons. At the very beginning, aptamers are oligonucleotides that are susceptible to degradation by nucleases. Secondly, the aptamer has a small diameter and is easily filtered by the kidneys and excreted quickly. Thirdly, aptamers are artificially synthesized non-natural nucleotides, may cause chemical toxicity or immunogenicity. Last but not the least, when chemically synthesized *in vitro* aptamers are used in an *in vivo* environment, their conformation may be altered, affecting their affinity with targets or pharmacokinetic characteristics (Zhou and Rossi, 2017). For aptamers exist defects in affinity, specificity, and stability, a variety of strategies have been taken to overcome these difficulties and improve the effectiveness of aptamers in clinical disease treatment. For example, chemical modification of aptamers after SELEX and SELEX *in vivo*. Common chemical modifications include the introduction of 2′-O-methyl RNA bases, 2′-fluoro, 2′-thiol, 2′-hydroxymethyl, amino (2′-NH2) or 2′-azido, and sugar-modified nucleotide analogs such as unlocked nucleic acid or locked nucleic acid. At the same time, aptamers have spiegelmers, and L-nucleotides cannot be recognized by nuclease or the immune system. These chemical modification methods improve the nuclease resistance of the aptamer. The combination of aptamers and macromolecules effectively delays renal clearance. The introduced macromolecules include polyethyleneglycol, cholesterol 3′-biotin-streptavidinbioconjugates liposomes, proteins, dendrimers, and inorganic nanoparticles (Röthlisberger and Hollenstein, 2018). People use different chemical modifications to improve the deficiency of aptamers, which will facilitate further development of aptamers in disease treatment. Although aptamers have not been approved for clinical cancer treatment, as a unique and novel anti-cancer drug, it will have outstanding development. Furthermore, Aptamers provide a strong impetus for the development of cancer treatment.

## Author Contributions

CL supervised the whole project. ZL consulted the literature and wrote the manuscript. PZ revised the manuscript. JH, YH, XF, and XC provided the technical support and professional manuscript. All authors contributed to the article and approved the submitted version.

## Conflict of Interest

The authors declare that the research was conducted in the absence of any commercial or financial relationships that could be construed as a potential conflict of interest.

## Abbreviations

BM: bone marrow
CAM-DR: cell adhesion-mediated drug resistance
ccRCC: clear cell renal cell carcinoma
CEA: carcinoembryonic antigen
CML: chronic myeloid leukemia
CRC: colorectal cancer
CXCL12: chemokine (C-X-C motif) ligand 12
E7: viral oncogenes
ECM: extracellular matrix
Ecto-ATP5B: plasma membrane-located ATP synthase
EGFR: epidermal growth factor receptor
eIF4E: eukaryotic translation initiation factor 4E
FDA: US Food and Drug Administration
Gal-1: galectin-1
HCC: hepatocellular carcinoma cells
HER2: human epidermal growth factor receptor 2
HPV: human papillomavirus
MM: multiple myeloma
MNK: MAP kinase-interacting kinase
PC: prostate cancer
PD-1: programmed cell death-1
PDGF-BB: platelet-derived growth factor-BB
PSMA: prostate-specific membrane antigen
SELEX: systematic evolution of ligands by exponential enrichment
TFs: transcription factors.
